# Expression patterns of seven key genes, including β-catenin, Notch1, GATA6, CDX2, miR-34a, miR-181a and miR-93 in gastric cancer

**DOI:** 10.1038/s41598-020-69308-0

**Published:** 2020-07-23

**Authors:** Narjes Jafari, Saeid Abediankenari, Zahra Hosseini-Khah, Seyed Mohammad Valizadeh, Zhila Torabizadeh, Ehsan Zaboli, Maryam Ghasemi, Hafez Fakheri, Vahid Hosseini, Ramin Shekarriz, Alireza Rafiei, Hossein Asgarian-Omran, Fatemeh Abedian

**Affiliations:** 1 Immunogenetics Research Center, Department of Immunology, Faculty of Medicine, Mazandaran University of Medical Sciences, Sari, Iran; 2 Diabetes Research Center, Faculty of Medicine, Mazandaran University of Medical Sciences, Sari, Iran; 3 Gut and Liver Research Center, Department of Internal Medicine, Faculty of Medicine, Mazandaran University of Medical Sciences, Sari, Iran; 4 Department of Pathology, Faculty of Medicine, Mazandaran University of Medical Science, Sari, Iran; 5 Gastrointestinal Cancer Research Center, Department of Internal Medicine, Faculty of Medicine, Mazandaran University of Medical Sciences, Sari, Iran; 6 Immunogenetics Research Center, Department of Pathology, Faculty of Medicine, Mazandaran University of Medical Science, Sari, Iran; 7 Molecular and Cell Biology Research Center, Department of Immunology, Faculty of Medicine, Mazandaran University of Medical Sciences, Sari, Iran; 8 Student Research Committee, Mazandaran University of Medical Sciences, Sari, Iran

**Keywords:** Cancer, Cell biology, Molecular biology, Molecular medicine

## Abstract

Gastric cancer (GC) is one of the most prevalent cancers and a major cause of cancer related mortality worldwide. Incidence of GC is affected by various factors, including genetic and environmental factors. Despite extensive research has been done for molecular characterization of GC, it remains largely unknown. Therefore, further studies specially conducted among various ethnicities in different geographic locations, are required to know the precise molecular mechanisms leading to tumorigenesis and progression of GC. The expression patterns of seven candidate genes, including β-catenin, Notch1, GATA6, CDX2, miR-34a, miR-181a, and miR-93 were determined in 24 paired GC tissues and corresponding non-cancerous tissues by quantitative Real-Time PCR. The association between the expression of these genes and clinicopathologic factors were also investigated. Our results demonstrated that overall mRNA levels of GATA6 were significantly decreased in the tumor samples in comparison with the non-cancerous tissues (median fold change (FC) = 0.3143; *P* = 0.0003). Overall miR-93 levels were significantly increased in the tumor samples relative to the non-cancerous gastric tissues (FC = 2.441; *P* = 0.0002). β-catenin mRNA expression showed a strong positive correlation with miR-34a (r = 0.5784; *P* = 0.0031), and miR-181a (r = 0.5652; *P* = 0.004) expression. miR-34a and miR-181a expression showed a significant positive correlation (r = 0.4862; *P* = 0.016). Moreover, lower expression of Notch1 was related to distant metastasis in GC patients with a borderline statistical significance (*p* = 0.0549). These data may advance our understanding of the molecular biology that drives GC as well as provide potential targets for defining novel therapeutic strategies for GC treatment.

## Introduction

Gastric cancer (GC) is one of the most prevalent cancers and the second leading cause of cancer-associated deaths worldwide. GC is a multifactorial disease affected by various genetic and environmental factors, including *Helicobacter pylori* infection, lifestyle, socioeconomic factors, dietary behavior, and aging^1,2^. This matter reflects an imperative need for conduction of studies among different geographic locations and ethnicities to detect multiple genes that are frequently dysregulated in cancers. Given that numerous genetic dysregulations may be implicated in tumor progression^3^, the study of combinations of several candidate genes, instead of a single marker, could be more helpful for the further understanding of cancer biology.

Β-catenin encoded by the CTNNB1 gene, is a multifunctional factor. It participates as a component of E-cadherin-catenin adhesion complex, and also is a transcriptional co-activator of Wnt/β-catenin signaling pathway. The abnormal expression of each components of the cadherin–catenin complex contributes to loss of adhesion between cells and may lead to carcinogenesis and metastasis^4–6^. In Wnt/β-catenin signaling pathway, transcriptional activation of target genes, such as c-jun, fra-1^7^, and c-myc^8^ proto-oncogenes, as well as many other genes involved in cell proliferation, metastasis, survival, dedifferentiation, and invasion^7,9^ could be occurred as a result of aberrant activities of β-catenin. Given the aforementioned statements, β-catenin may contribute to carcinogenesis with two contradictory roles: as a tumor suppressor involved in cadherin-mediated cell to cell adhesion system, and as a proto-oncogene involved in the Wnt/β-catenin pathway. Thus, assessment of aberrant expression of β-catenin is a worthy option that needs to be considered in further cancer researches.

The Notch signaling pathway is implicated in a variety of cellular processes including proliferation, differentiation, apoptosis, cell fate determination and maintenance of stem cell^10,11^. In mammals, there are four Notch receptors (Notch1-4). After ligand-receptor binding in the Notch signaling pathway, the intracellular domain of Notch receptor is cleaved and subsequently translocated into the nucleus to activate the expression of downstream target genes. The Notch receptors act either as oncogenes or tumor suppressors depending on cellular context and cross talk with other pathways. For example, an oncogenic function of Notch1 in T-cell acute lymphoblastic leukemia (T-ALL) and a tumor suppressive role of Notch1/2 in small cell lung cancer (SCLC) were reported^12^.

GATA transcription factors regulate the expression of specific genes implicated in cell lineage development and differentiation and contain six members in vertebrates. GATA1/2/3 are mainly expressed in the hematopoietic cell lineages and GATA4/5/6 are expressed in tissues with mesodermal and endodermal origins^13,14^. For GATA6, both tumor suppressor and proto-oncogene activities have been reported. Down-regulation or loss of expression of GATA6 was reported in ovarian cancer^13,15^, adrenocortical tumor^16^, lung adenocarcinoma^17^, and astrocytoma^18^. In addition to the tumor-suppressive activity, data also suggests that GATA6 has an oncogenic function. Although there are some conflicting reports^19^, GATA6 expression is up-regulated mainly in digestive system cancers, such as pancreatic cancer^20–22^, esophageal adenocarcinoma^23,24^, colon/colorectal cancer^25–27^, and in liver metastatic samples from colorectal cancer patients^26^. Activation of canonical wnt signaling pathway is one mechanism by which GATA6 promotes carcinogenesis^22^.

Nuclear transcription factor CDX2 is a member of the caudal-related homeobox family and specifically expressed in the epithelium of the intestines and colon. It has been shown to be an important regulator in differentiation, proliferation and maintenance of intestinal epithelial cells^28–32^. Reduced expression of CDX2 was reported to be associated with colorectal cancer^33^.

MicroRNAs (miRNAs, miRs) are small noncoding RNAs, which regulate the expression of genes at the post-transcriptional level by targeting the 3ʹ-untranslated regions (3ʹ-UTR) of mRNAs^34^. Accumulating evidence indicates that miRNAs are aberrantly expressed in cancers and function as oncogenes or tumor suppressor genes^35,36^.

miR-34 family is involved in cell cycle arrest, senescence and apoptosis. Previous studies have revealed that miR-34a mainly acts as tumor suppressor and its expression is down-regulated or lost in different types of cancers^34^. Notably, an oncogenic function for miR-34a was demonstrated in liver tumors with β-catenin overactivation^37^.

miR-181a has roles in crucial cellular events, including development, differentiation, hematopoiesis, immune modulation and inflammatory responses^38–41^. Nonetheless, miR-181a showed tumor suppressive effects in a variety of cancers, including oral squamous cell carcinoma^42^, leukemia^43^, and glioblastoma^36^. In contrast, it was identified as an oncogene and its up-regulation was observed in various cancers, including cervical cancer^44^, hepatocellular carcinoma^45^, and breast cancer^46^. This dichotomy in miR-181a function may be due to targeting different oncogenes or tumor suppressor genes depending on the tissue or cellular microenvironment^47^, demonstrating a tumor-specific role of miR-181a in tumorigenesis^48^.

miR-93 derives from the pro-oncogenic miR-106b-25 cluster. This cluster encodes three miRNAs, including miR-106b, miR-93 and miR-25, which functions in apoptosis, cell cycle progression, proliferation and differentiation processes^49^. miR-93 acts as an oncogene and is overexpressed in several type of cancers, such as hepatocellular carcinoma^50^, lung cancer^51^, osteosarcoma^52^ and head and neck squamous cell carcinoma^53^. In contrast, miR-93 was found to be suppressor in ovarian carcinoma^54^. Furthermore, there is an opposite data related to the role of miR-93 in breast cancer. In Singh et al.^55^ study, the oncogenic function of miR-93 was verified, which promoted breast carcinogenesis, whiles, Xiang et al.^56^ showed a tumor suppressor action of miR-93, which inhibited the epithelial-mesenchymal transition (EMT) of breast cancer cells. This data and other similar findings make us more cautious about the use of miRNAs/anti-miRNAs in cancer treatment.

According to the above mentioned information, these seven candidate genes need to be characterized more precisely in cancerogenesis.

In the present study, we evaluated the expression levels of the above mentioned genes by Real- Time PCR (RT-PCR) between paired samples of GC and normal tissues, in order to determine the association of aberrantly expressed genes with clinicopathologic features of patients. The results of this study will be useful to clarify the molecular pathogenesis underlying the development of GC, and can be helpful to identify suitable targets for diagnosis and treatment of the disease.

## Results

### Patients and tumor samples

Gastric carcinoma tissues and normal adjacent gastric tissues were obtained from 24 patients undergoing endoscopy for diagnostic purposes. Clinico-pathological data was shown in Table 1. Briefly, at the time of diagnosis, the age of patients (6 female/18 male) ranged from 48 to 89 years (mean 70.25 years). The gastric carcinomas were classified as intestinal (n = 15) and diffuse histological types (n = 9) according to Lauren system^57^. Intestinal type tumors were graded into poorly, moderately or well differentiated. Well- and moderately differentiated tumors were grouped together for purposes of statistical analysis. All diffuse cancers were classed as poorly differentiated. In the other words, 10 poorly, and 14 differentiated tumor tissues were included in our study. Given that the classification of tumor samples based on grading was rather similar to that of histological types (intestinal and diffuse types), our presented data related to altered gene expression in histological types reflects the changes also for two groups of cancer grades.

**Table 1:** Patients and histopathological characteristics of cancerous tissues.

Case number	Age/sex	Lauren classification	Histologic grade	Stage	Distant metastasis
T3	71/M	Intestinal	MD	IIIb	–
T4	62/M	Diffuse	PD	II	ND
T5	63/M	Intestinal	MD	IV	+
T6	66/F	Diffuse	PD	ND^a^	–
T8	79/F	Intestinal	MD	III	–
T9	82/M	Intestinal	WD	ND	ND
T10	80/M	Intestinal	MD	IV	+
T11	80/M	Diffuse	PD	At least III	ND
T12	82/M	Intestinal	WD	IV	+
T13	89/M	Intestinal	MD	IV	+
T14	68/M	Intestinal	PD	IV	+
T15	84/M	Intestinal	MD	At least III	ND
T17	64/F	Diffuse	PD	At least III	ND
T20	69/M	Diffuse	PD	IIIa	–
T22	53/M	Intestinal	WD/MD	IV	+
T23	48/F	Diffuse	PD	III	–
T24	69/M	Intestinal	WD	Ib	–
T25	65/M	Diffuse	PD	ND	ND
T26	61/F	Diffuse	PD	IV	+
T27	80/M	Intestinal	MD	IV	+
T28	75/M	Intestinal	MD/WD	IIIb	–
T29	59/M	Diffuse	PD	IV	+
T30	71/F	Intestinal	MD	IV	ND
T32	66/M	Intestinal	MD	At least III	ND

*M* male, *F* female, *WD* well-differentiated, *PD* poorly differentiated, *MD* moderately differentiated, *ND* not determined.

^a^Additional information was not available because of some reasons such as elderly patients rarely underwent surgery and some patients went to another city for medical care.

### Expression patterns of candidate genes in gastric tumor and non-tumor tissues

**Figure 1:**
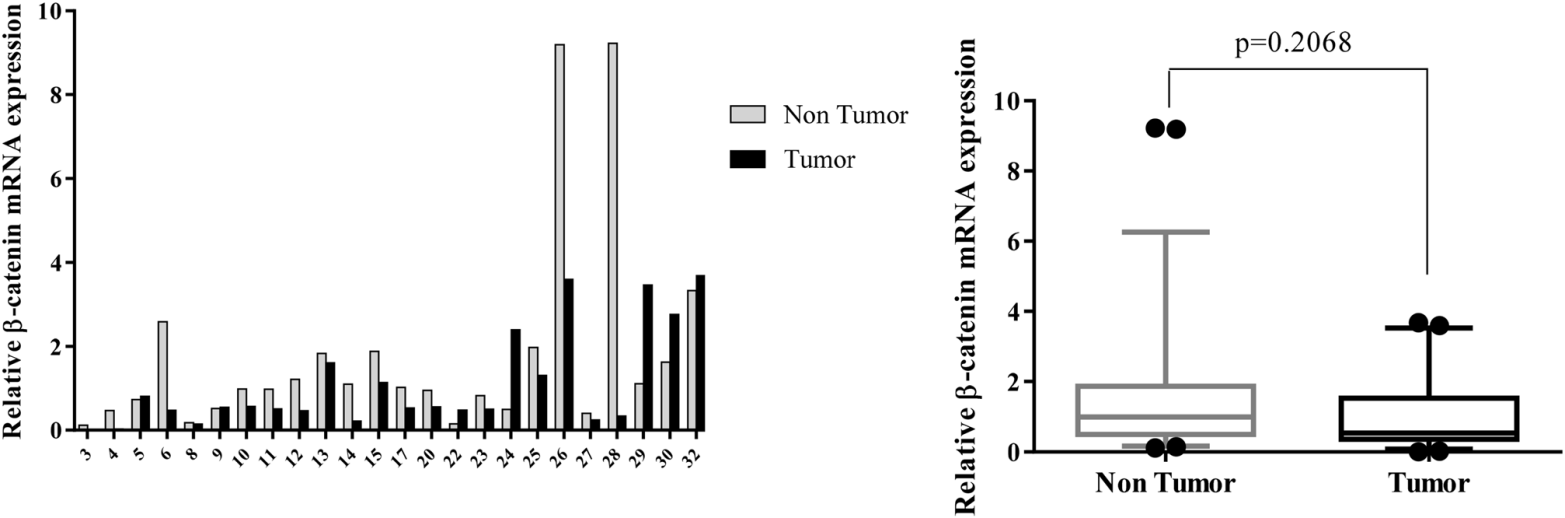
Quantitative RT-PCR analysis of β-catenin levels in 24 paired gastric carcinoma and adjacent normal tissues. Left curve: Each column indicates the fold change of expression value (2^−∆∆Ct^) of the genes in individual tumor (black column) or control (gray column) tissue normalized by the median expression levels (∆Ct) of the all control tissues, as described in “Methods” section. Numbers on the bottom axis correspond to case numbers in Table 1. Right curve: Overall RNA expression levels in tumor samples compared to non-tumor samples. Quantitative RT-PCR data were presented as box plots showing the median of overall RNA expression in tumor samples relative to non-tumor samples. The lines inside the boxes denote the medians. The whiskers of box plots: 10–90%. Decreased β-catenin mRNA levels in the tumor tissues were not statistically significant (median FC = 0.535). Statistical differences were evaluated between two groups by non-parametric Mann–Whitney test.

**Figure 2:**
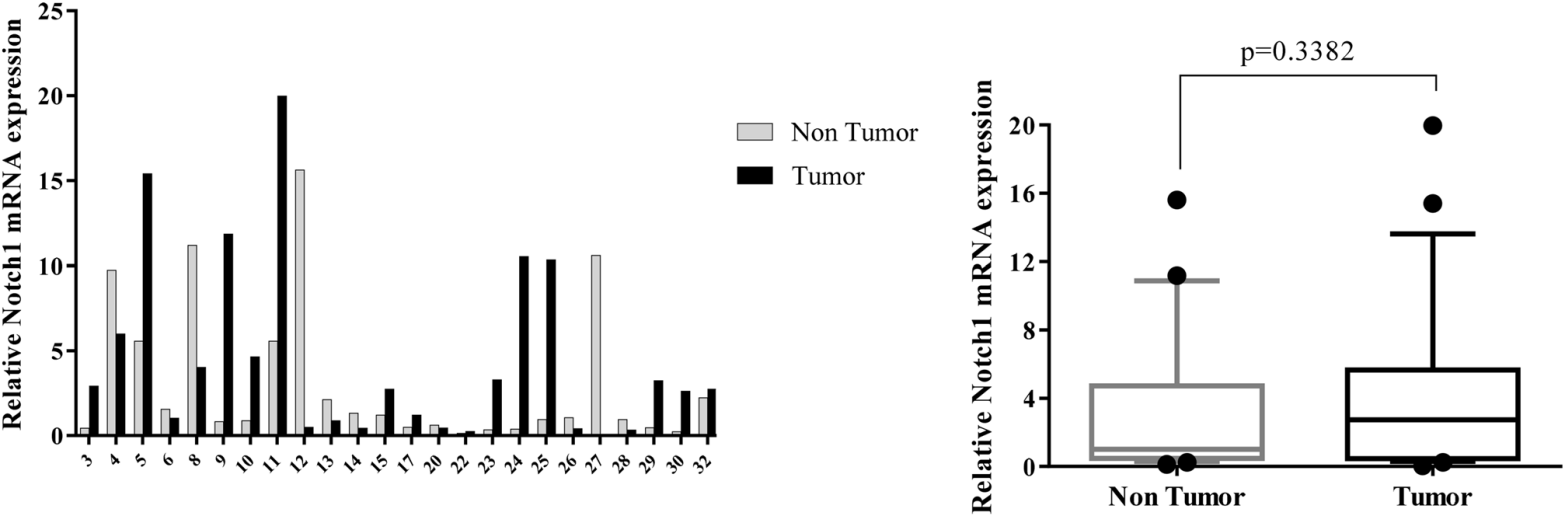
Quantitative RT-PCR analysis of Notch1 expression levels in 24 paired gastric carcinoma and adjacent normal tissues. Left curve: Each column indicates the fold change of expression value (2^−∆∆Ct^) of the genes in individual tumor (black column) or control (gray column) tissue normalized by the median expression levels (∆Ct) of the all control tissues, as described in “Methods” section. Numbers on the bottom axis correspond to case numbers in Table 1. Right curve: Overall RNA expression levels in tumor samples compared to non-tumor samples. Quantitative RT-PCR data were presented as box plots showing the median of overall RNA expression in tumor samples relative to non-tumor samples. The lines inside the boxes denote the medians. The whiskers of box plots: 10–90%. Increased RNA levels of Notch1 in the tumor tissues were not statistically significant (median FC = 2.742). Statistical differences were evaluated between two groups by non-parametric Mann–Whitney test.

**Figure 3:**
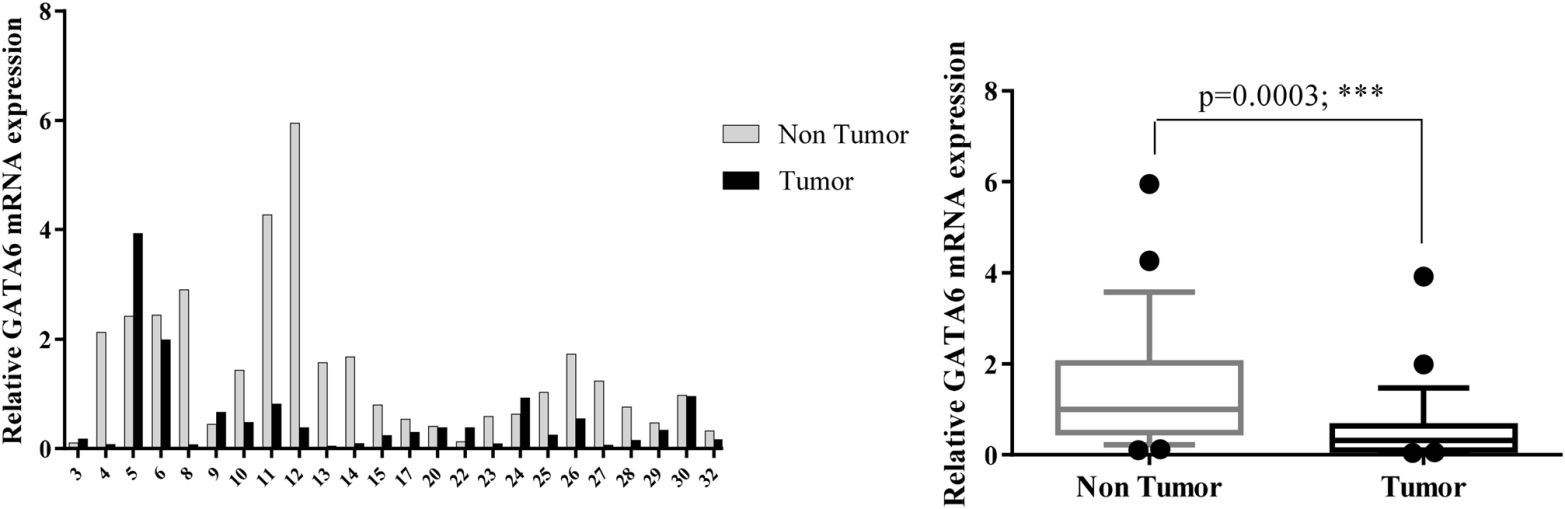
Quantitative RT-PCR analysis of GATA6 expression levels in 24 paired gastric carcinoma and adjacent normal tissues. Left curve: Each column indicates the fold change of expression value (2^−∆∆Ct^) of the genes in individual tumor (black column) or control (gray column) tissue normalized by the median expression levels (∆Ct) of the all control tissues, as described in “Methods” section. Numbers on the bottom axis correspond to case numbers in Table 1. Right curve: Overall RNA expression levels in tumor samples compared to non-tumor samples. Quantitative RT-PCR data were presented as box plots showing the median of overall RNA expression in tumor samples relative to non-tumor samples. The lines inside the boxes denote the medians. The whiskers of box plots: 10–90%. Overall mRNA levels of GATA6 were significantly decreased in the tumor samples relative to the non-cancerous gastric tissues (median FC = 0.3143). Statistical differences were evaluated between two groups by non-parametric Mann–Whitney test. *** indicate *P* < 0.001; FC: fold change.

**Figure 4:**
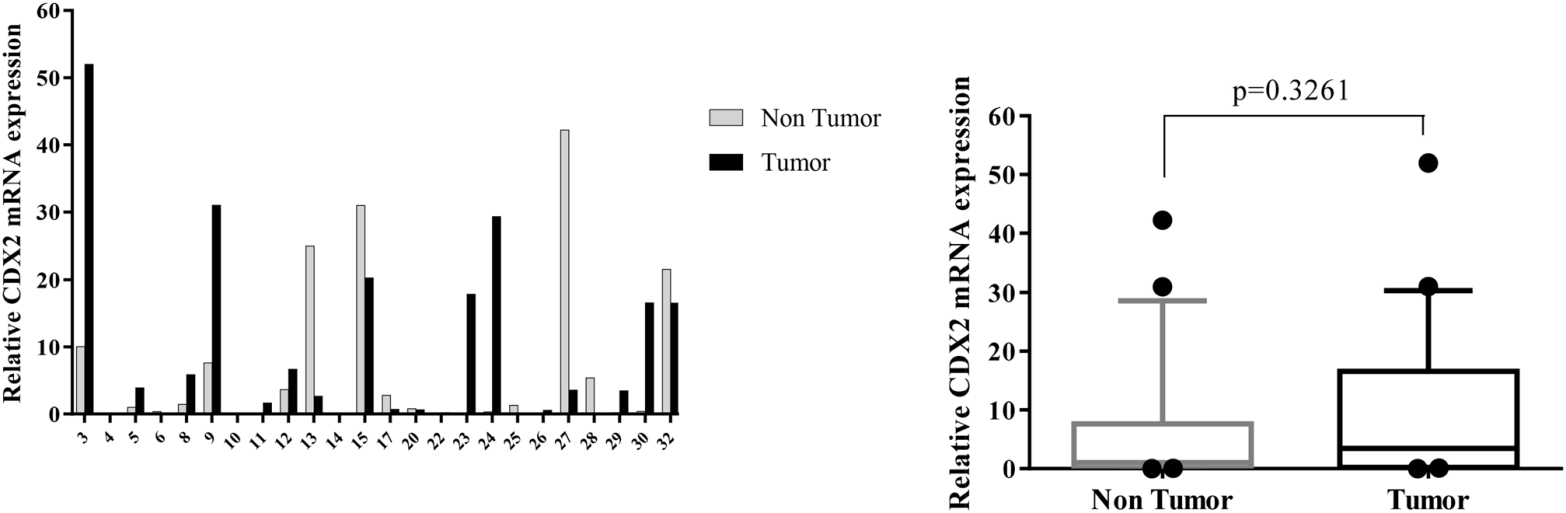
Quantitative RT-PCR analysis of CDX2 expression levels in 24 paired gastric carcinoma and adjacent normal tissues. Left curve: Each column indicates the fold change of expression value (2^−∆∆Ct^) of the genes in individual tumor (black column) or control (gray column) tissue normalized by the median expression levels (∆Ct) of the all control tissues, as described in “Methods” section. Numbers on the bottom axis correspond to case numbers in Table 1. Right curve: Overall RNA expression levels in tumor samples compared to non-tumor samples. Quantitative RT-PCR data were presented as box plots showing the median of overall RNA expression in tumor samples relative to non-tumor samples. The lines inside the boxes denote the medians. The whiskers of box plots: 10–90%. Increased RNA levels of CDX2 in the tumor tissues were not statistically significant (median FC = 3.47). Statistical differences were evaluated between two groups by non-parametric Mann–Whitney test.

**Figure 5:**
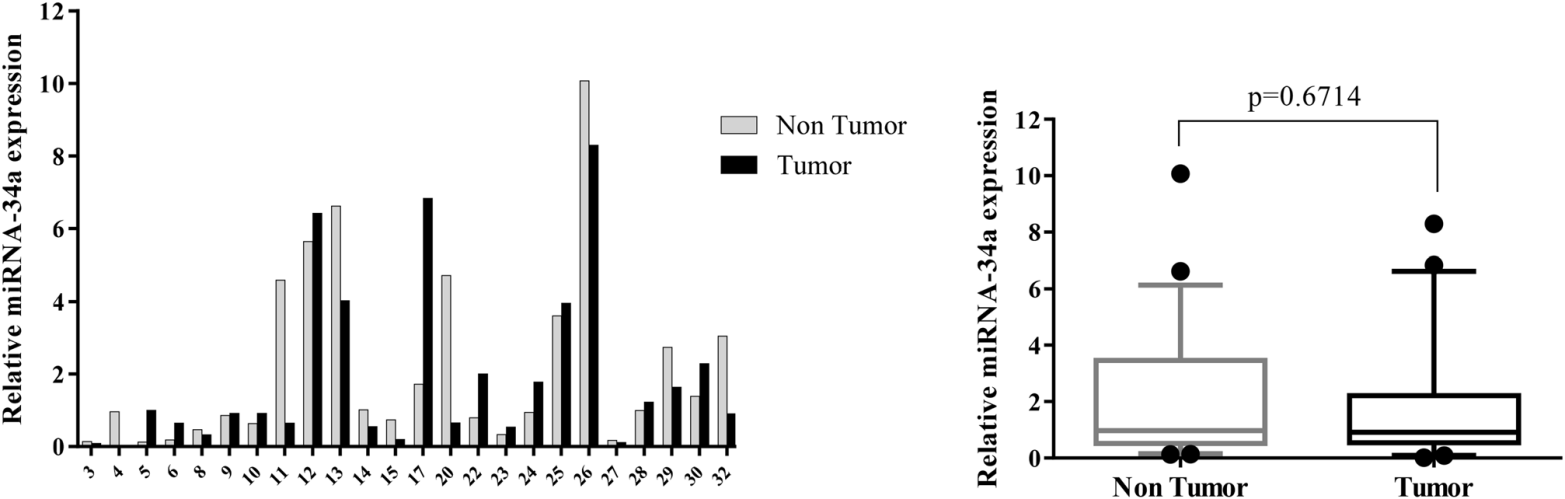
Quantitative RT-PCR analysis of miR-34a expression levels in 24 paired gastric carcinoma and adjacent normal tissues. Left curve: Each column indicates the fold change of expression value (2^−∆∆Ct^) of the genes in individual tumor (black column) or control (gray column) tissue normalized by the median expression levels (∆Ct) of the all control tissues, as described in “Methods” section. Numbers on the bottom axis correspond to case numbers in Table 1. Right curve: Overall RNA expression levels in tumor samples compared to non-tumor samples. Quantitative RT-PCR data were presented as box plots showing the median of overall RNA expression in tumor samples relative to non-tumor samples. The lines inside the boxes denote the medians. The whiskers of box plots: 10–90%. Decreased levels of miR-34a in the tumor tissues were not statistically significant (median FC = 0.915). Statistical differences were evaluated between two groups by non-parametric Mann–Whitney test.

**Figure 6:**
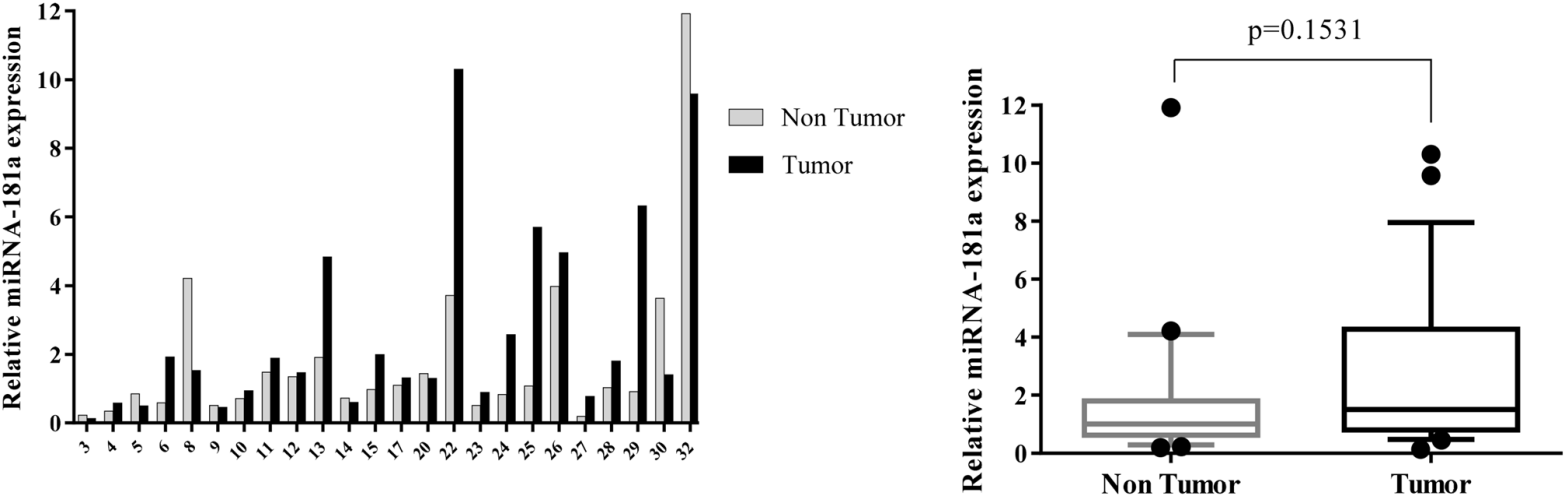
Quantitative RT-PCR analysis of miR-181a expression levels in 24 paired gastric carcinoma and adjacent normal tissues. Left curve: Each column indicates the fold change of expression value (2^−∆∆Ct^) of the genes in individual tumor (black column) or control (gray column) tissue normalized by the median expression levels (∆Ct) of the all control tissues, as described in “Methods” section. Numbers on the bottom axis correspond to case numbers in Table 1. Right curve: Overall RNA expression levels in tumor samples compared to non-tumor samples. Quantitative RT-PCR data were presented as box plots showing the median of overall RNA expression in tumor samples relative to non-tumor samples. The lines inside the boxes denote the medians. The whiskers of box plots: 10–90%. Increased level of miR-181a in the tumor tissues were not statistically significant (median FC = 1.5). Statistical differences were evaluated between two groups by non-parametric Mann–Whitney test.

**Figure 7:**
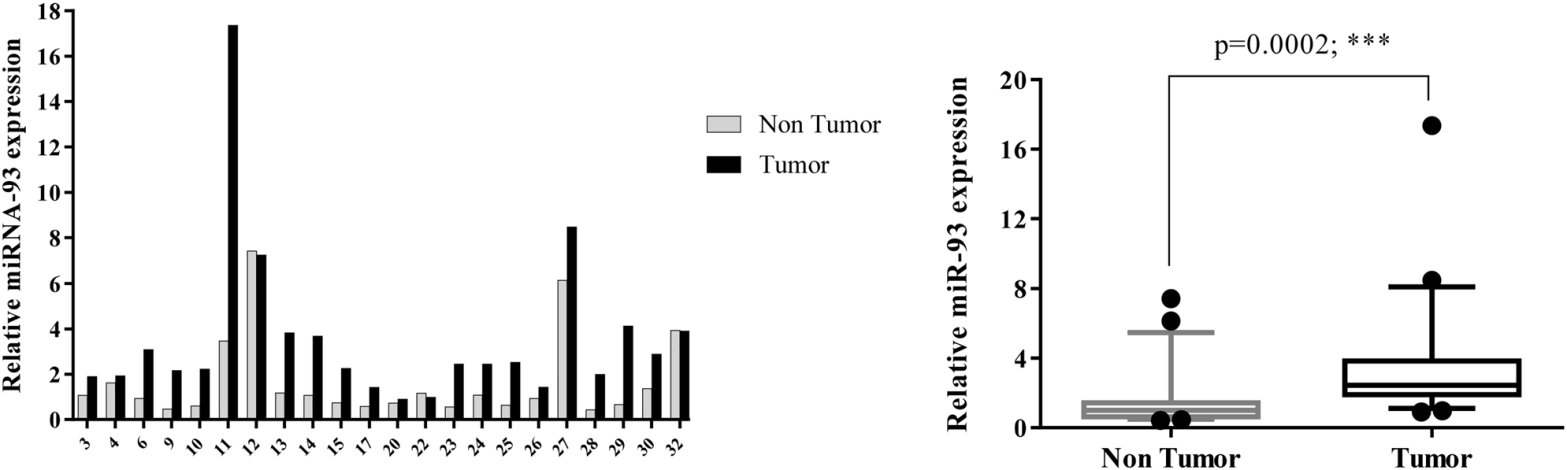
Quantitative RT-PCR analysis of miR-93 expression levels in 22 paired gastric carcinoma and adjacent normal tissues. Left curve: Each column indicates the fold change of expression value (2^−∆∆Ct^) of the genes in individual tumor (black column) or control (gray column) tissue normalized by the median expression levels (∆Ct) of the all control tissues, as described in “Methods” section. Numbers on the bottom axis correspond to case numbers in Table 1. Right curve: Overall RNA expression levels in tumor samples compared to non-tumor samples. Quantitative RT-PCR data were presented as box plots showing the median of overall RNA expression in tumor samples relative to non-tumor samples. The lines inside the boxes denote the medians. The whiskers of box plots: 10–90%. Overall miR-93 levels were significantly increased in the tumor tissues relative to the non-cancerous gastric tissues (median FC = 2.441). Statistical differences were evaluated between two groups by non-parametric Mann–Whitney test. *** indicate *P* < 0.001; FC: fold change.

Individual samples of RNA were evaluated for the transcript levels of seven genes, including β-catenin, Notch1, GATA6, CDX2, miR-34a, miR-181a, and miR-93; and fold change of gene expression was calculated. Differential expression in tumor versus corresponding non-tumor tissues in each patient was shown as Figs. 1, 2, 3, 4, 5, 6 and 7 (left). Overall RNA expression levels in tumor samples compared to non-tumor samples were also presented in Figs. 1, 2, 3, 4, 5, 6 and 7 (right). The qRT-PCR analysis showed that overall mRNA levels of GATA6 were significantly decreased in the tumor samples relative to the adjacent non-cancerous tissues (median FC = 0.3143; *P* = 0.0003). Overall miR-93 levels were significantly increased in the tumor samples relative to the non-cancerous gastric tissues (median FC = 2.441; *P* = 0.0002). Overall RNA levels of β-catenin and miR-34a were decreased in the tumor samples in comparison with non-cancerous tissues (median FC = 0.535 and 0.915, respectively), but these differences were not statistically significant (*P* = 0.2068, *P* = 0.6714, respectively). In addition, overall RNA levels of Notch1, CDX2 and miR-181a were increased in tumor samples relative to non-cancerous tissues (median FC = 2.742, 3.47 and 1.5, respectively). However, these differences were not statistically significant (*P* = 0.3382, *P* = 0.3261, *P* = 0.1531, respectively) (Figs. 1, 2, 3, 4, 5, 6, 7 right).

### Expression levels of candidate genes between intestinal- and diffuse-type GCs

**Figure 8:**
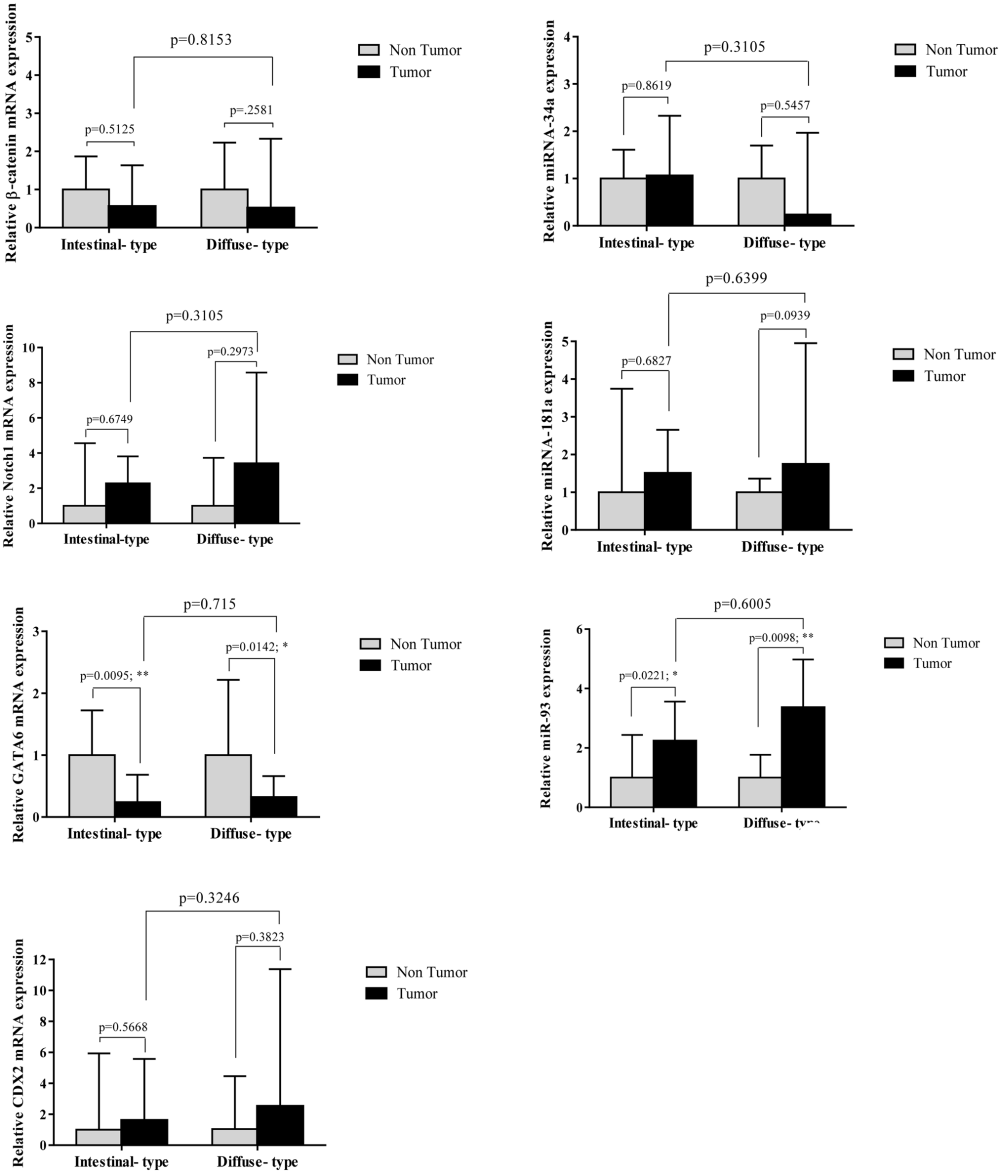
Comparison of RNA expression levels between intestinal- and diffuse- type gastric cancers. Statistical differences were evaluated between two groups by non-parametric Mann–Whitney test. Data were presented as the median with interquartile range. * and ** indicate *P* < 0.05 and *P* < 0.01, respectively.

The RNA (mRNA or miRNA) expression of the candidate genes was compared between 15 intestinal-type and 9 diffuse-type cancer tissues. As mentioned above, expression levels of GATA6 mRNA were significantly decreased in tumor samples relative to the corresponding non-tumor samples. Further analysis showed the frequent reduction of GATA6 expression in intestinal-type tumors than the diffuse- type tumors when compared to their corresponding non-tumor tissues (*p* = 0.0095 and *p* = 0.0142, respectively). However, significant statistical difference was not detected between these two cancer types in GATA6 expression (*p* = 0.715) (Fig. 8). A significant increased level of miR-93 expression was also observed in intestinal- and diffuse-type cancers relative to the corresponding non-tumor samples (*p* = 0.0.221 and *p* = 0.0.098, respectively) but found no statistically significant difference in miRNA level between the two histological types (*p* = 0.6005). Furthermore, expression levels of miR-34a were decreased specifically in diffuse-type cancers without significant statistical differences compared to the intestinal-type (*p* = 0.3105) and also to non-tumor samples (*p* = 0.5455). Additionally, expression levels of β-catenin, Notch1, CDX2, and miR-181a showed no significant difference between intestinal- and diffuse-type cancers (*p* = 0.8153, 0.3105, 0.3246, 0.6399, respectively), although the expression of Notch1 and CDX2 were somewhat up-regulated in diffuse-type cancer (Fig. 8).

### Correlation between the expressions of candidate genes in GC

**Figure 9:**
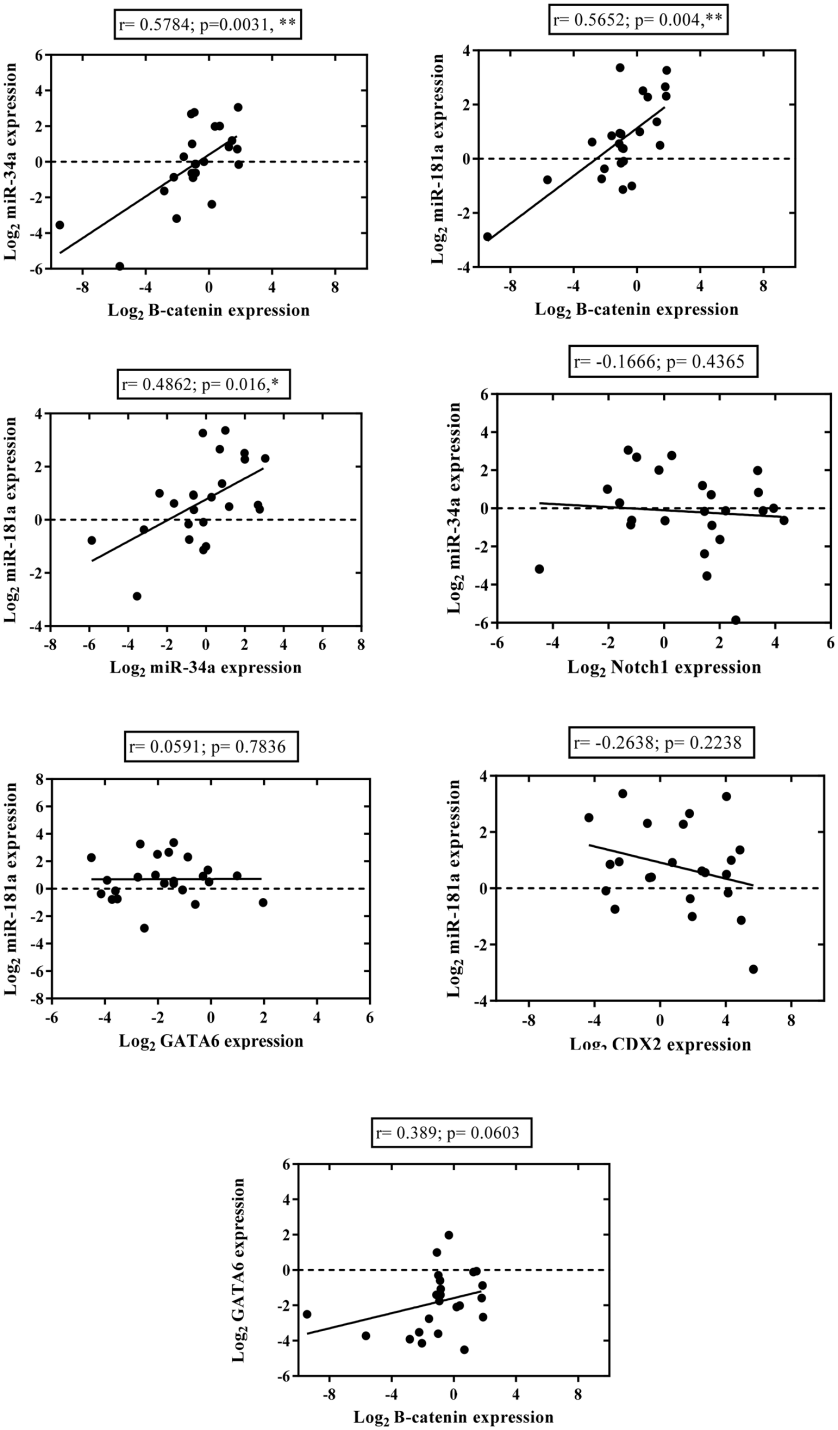
Correlation patterns of the genes expression in gastric cancer tissues were examined using quantitative RT-PCR. Curves exhibit the correlations between the expression levels of the genes in total gastric cancer tissues. Expression levels are given as Log_2_^Fold Change^ (or −∆∆ Ct). R and *P* values were calculated by Spearmanʼs correlation method. * and ** indicate *P* < 0.05 and *P* < 0.01, respectively.

With regards to the concomitant expression of the seven RNAs in total tumor samples, β-catenin mRNA expression showed a strong positive correlation with miR-34a (r = 0.5784; *P* = 0.0031), and miR-181a (r = 0.5652; *P* = 0.004) expression (Fig. 9). In addition, miR-34a and miR-181a expression showed a significant positive correlation (r = 0.4862; *P* = 0.016) (Fig. 9). Also, a positive correlation was detected between β-catenin and GATA6 expression. However, the statistical significance of this finding was borderline (r = 0.389; *p* = 0.0603) (Fig. 9). When we assessed the expression of the genes with significant relationships between two histological types, we found some minor differences in the correlation patterns. A significant positive correlation between β-catenin with miR-34a and miR-181a were detected only in diffuse-type cases (r = 0.8667; *p* = 0.0045, and r = 0.7167; *p* = 0.0369, respectively) (Supplementary Fig. 1). Although β-catenin expression was also positively associated with miR-34a and miR-181a in intestinal-type cases, but these correlations were not statistically significant (r = 0.4629; *p* = 0.0838, and r = 0.4821; *p* = 0.0711) (Supplementary Fig. 1). Furthermore, a remarkably positive correlation was detected between miR-34a and miR-181a in each histological type (r = 0.6333; *p* = 0.076, and r = 0.4147; *p* = 0.1249, respectively for diffuse- and intestinal-type) (Supplementary Fig. 1), which was not statistically significant.

### Association of the genes under investigation with distant metastasis

**Figure 10:**
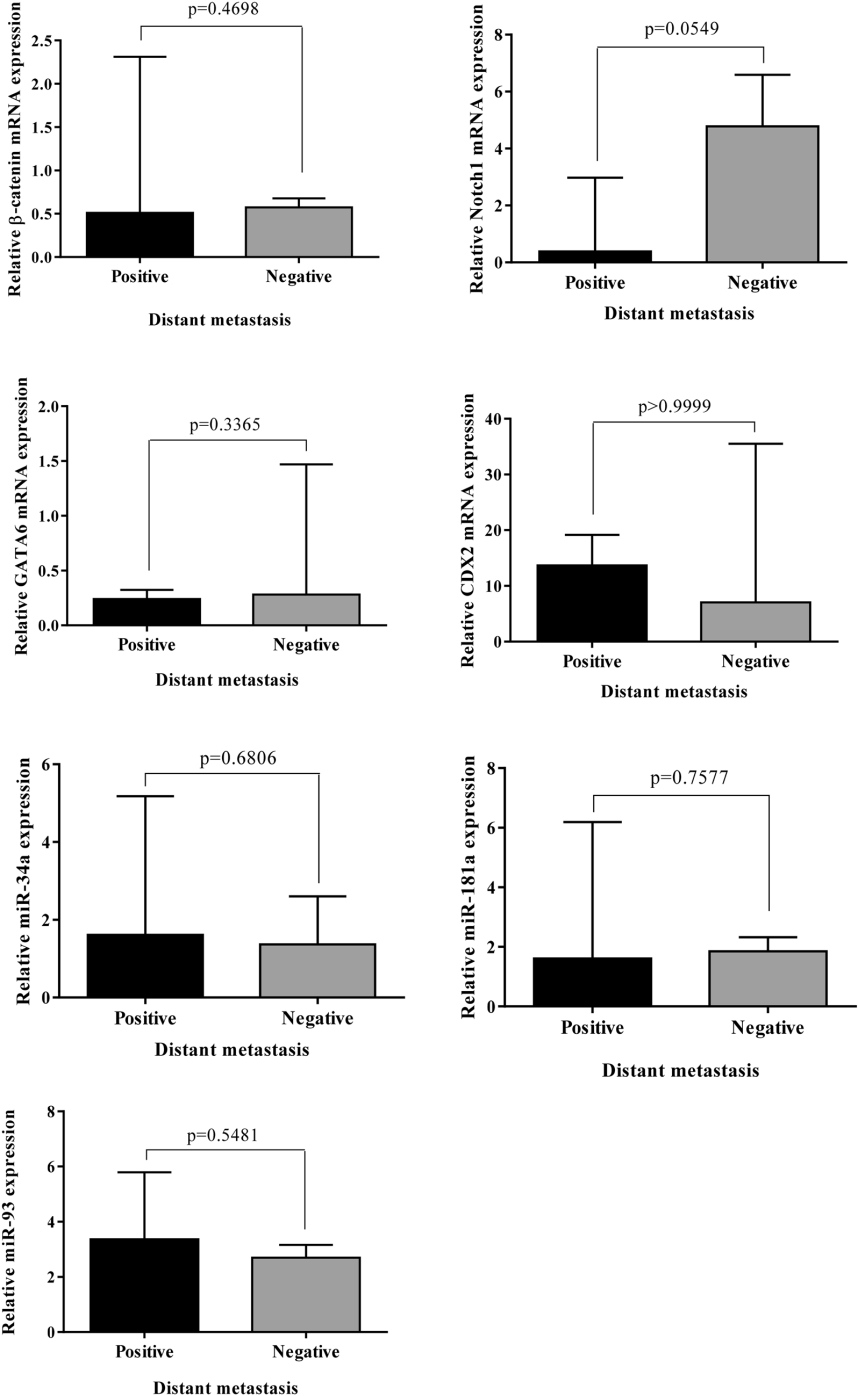
Association between the expression levels of the genes and distant metastasis in gastric cancer patients. The low expression of Notch1 in gastric cancer tissues showed a borderline significant association with distant metastasis.

We further investigated whether expression levels of these genes are associated with distant metastasis in GC. We compared the RNA expression between 9 patients with distant metastasis and 7 patients without metastasis. According to the qRT-PCR analysis, lower expression of Notch1 was detected in patients with distant metastasis. The difference of Notch1 expression between the two groups of patients (with and without metastasis) was borderline significant (*p* = 0.0549), thus, the lower expression level of Notch1 was relatively associated with distant metastasis in GC. The expression levels of other genes were not obviously associated with distant metastasis (Fig. 10).

## Discussion

In this study, the expression profiles of seven genes, including β-catenin, Notch1, GATA6, CDX2, miR-34a, miR-181a, and miR-93 were determined in GC tissues and corresponding noncancerous tissues in order to find characteristic changes associated with pathogenesis and progression of GC.

We compared the ratios of β-catenin RNA expression fold change in tumor tissue to that in corresponding non tumor tissue in each patient, and found that 29.1% of patients showed a decreased level (FC T/N < 0.5) of β-catenin expression, whereas, 12.5% of them showed an elevated level (FC T/N > 2) of expression (Fig. 1). An overall analysis showed lower level of β-catenin expression in tumor tissues relative to non-tumor tissues that was statistically non-significant (Fig. 1). With reference to previous studies, we found that transcription levels of β-catenin gene in GC tissues has not been convincingly investigated by RT-PCR. However, studies on abnormalities of β-catenin protein expression, specially using immunohistochemistry technique, are noticeable. In a study by Jawhari et al.^6^, loss of membranous β-catenin protein expression was reported in 58% of diffuse-type GCs and in 38% of intestinal-type GCs. In Ramesh et al. study, ectopic intracellular expression of β-catenin protein was relatively rare. Nonetheless, reduced or loss of membranous β-catenin protein expression was found in 83.4% of diffuse and in 28.6% of intestinal type cancers^58^. In another study, the quantitative analysis of β-catenin protein levels in GC samples versus their matched normal gastric mucosa by western blot did not reveal any significant difference^5^. However, in contrast with our findings, the study of Ebert et al. revealed that overall mRNA levels of β-catenin were significantly increased in GC samples. In addition, increased β-catenin mRNA levels were reported more frequent in intestinal-type GCs than diffuse-type GCs^4^. These inconsistence results may be due to different mechanisms that disturb β-catenin expression such as mutations of the β-catenin gene—although β-catenin gene mutations seems to be infrequent in GCs^4,59–61^—, mutations of APC gene^4,5^, or other components involved in Wnt pathway^59^, hypermethylation of the β-catenin promoter^5^, and hypermethylation of the APC promoter^62^.

We detected an increased level (FC T/N > 2) of Notch1 expression in 50% of patients and a decreased level (FC T/N < 0.5) of that in 29.1% patients (Fig. 2). However, we found no significant alteration in overall Notch1 expression between GC tissues and non-cancerous tissues (Fig. 2). Little is known about the dysregulated expression of Notch1 in GC tissues, as well as, its correlation with other genes and clinical features. Yeh et al. study showed that 63.3% of GC patients expressed Notch1 protein in cancer tissues. Furthermore, the activation of Notch1 signaling promoted tumor progression of stomach adenocarcinoma SC-M1 cells through induction of COX-2 (cyclooxygenase-2 (expression^11^. Another study reported that the activated form of Notch1 (N1IC) elevated the progression of several human GC cell lines through STAT3 and Twist expression^63^. N1IC also enhanced GC progression through miR-151-5p^64^.

Noteworthy, the results of our study suggested that down-regulation of Notch1 may be associated with the potential for distant metastases in GC. The statistical significance of this finding was borderline (*p* = 0.0549), possibly due to the small size of sample or extensive variations in expression levels of Notch1 between individual patients.

In our study, 58.3% of patients showed a decreased level (FC T/N < 0.5) of GATA6 expression, whereas, 4.1% of them showed an elevated expression level (FC T/N > 2) of that (Fig. 3). We found that overall mRNA levels of GATA6 were significantly decreased in the tumor samples in comparison with non-cancerous gastric tissue (*P* = 0.0003) (Fig. 3).

Little is known about deregulated expression of GATA6 in GC. In accordance with our study, Liu et al. reported that the expression of GATA6 was significantly decreased in metastatic GC cells and tissues. In addition, they found that overexpression of GATA6 inhibited GC cell migration, invasion and metastasis in vitro and in vivo^65^.

In contrast with our findings, Sulahian et al. reported that GATA6 gene is amplified or overexpressed in gastric, esophageal and pancreatic adenocarcinomas. They further found that depletion of GATA6 impairs gastrointestinal cancer cell growth and induces cell cycle arrest in G2/ M phase^66^. Thus, the exact role of GATA6 in gastric carcinogenesis has remained controversial due to the limited and inconsistent reports.

We determined an elevated level (FC T/N > 2) of CDX2 expression in 52.1% of GC patients and a decreased level (FC T/N < 0.5) of that in 26% of patients (Fig. 4). Furthermore, significant differences were no observed in increased level of CDX2 mRNA between total GC tissues and non-cancerous tissues (Fig. 4). The potential roles of CDX2 in the gastric carcinogenesis and progression are also complex and remain unclear. In spite of some conflicting studies^29,67,68^, an overall experimental insight provided from previous studies is that CDX2 acts as tumor suppressor in GC^31,32^. Kim et al. showed that CDX2 mRNA expression was considerably higher in gastric tumor tissues than non-tumor tissues due to DNA hypomethylation. Consistent with our result, they also found no statistically significant difference in CDX2 mRNA levels between two GC types^69^. As well as, in Almedia et al.^28^ study, CDX2 protein expression was not detected in the nuclei of normal mucosa cells adjacent to gastric carcinomas, while that was observed in 54% of cases, regardless of GC histological types. However, inappropriate activation of CDX2, as an intestine-specific gene, in gastric may be involved in intestinal differentiation, a pathway towards the gastric carcinogenesis^28,67,68^. Thus, due to inconsistent results, the precise role of CDX2 overexpression in gastric carcinogenesis and malignancy remains to be further clarified.

In our study, a decreased level (FC T/N < 0.5) of miR-34a expression was detected in 25% of GC patients and an increased level (FC T/N > 2) of that was observed in 8.3% of patients (Fig. 5). Although decreased level of miR-34a was not statistically significant between two GC types, this reduction was more frequent in diffuse-type cases than intestinal-type cases (median miR-34a expression FCs were 0.237 and 1.064, respectively for diffuse-and intestinal-type) (Fig. 8). According to qRT-PCR analysis, numerous studies reported that the levels of miR-34a expression were significantly decreased in the GC patients^35,70^. Meanwhile, miR-34a expression levels were detected lower in patients with metastasis than in patients without metastasis^35^. However, there are controversial studies that reported a significant up-regulation of miR-34a level in GC tissues compared to normal gastric tissues^71,72^. It is noteworthy that these studies used the microarray analysis to determine the expression profile of miRNA in GC and normal tissues without further validation of miR-34a expression by qRT-PCR.

We detected an increased level (FC T/N > 2) of miR-181a expression in 33.3% of patients and a decreased level (FC T/N < 0.5) of that in 8.3% patients (Fig. 6). The overall difference in miR-181a expression was not statistically significant between GC tissues and non-cancerous tissues (Fig. 6). Indeed, miR-181a-5p was found as an onco-miRNA promoting cell proliferation, metastasis, invasion and EMT in GC cell lines^47,73–75^. Although there are numerous studies reporting the up-regulated expression of miR-181a-5p in GC tissues^48,75,76^, in controversial study, Lin et al. reported that the expression of miR-181a in GC tissues was significantly lower than in adjacent tissues. Their results suggested that miR-181a acts as a tumor suppressor and its down-regulation may involve in the progression and metastasis of GC^77^. Therefore, the molecular mechanisms by which miR-181a mediate the pathogenesis of GC still need to be further elucidated.

In the present study, an elevated level (FC T/N > 2) of miR-93 expression was detected in 63.6% of patients (Fig. 7). Overall miR-93 levels were strongly increased in the tumor samples relative to the non-cancerous gastric tissues (*P* = 0.0002) (Fig. 7). Several studies reported the higher expression of miR-93 in GC tissues compared with the noncancerous tissues, suggesting that miR-93 functions as a promoter for tumor progression in GC patients. In vitro and in vivo studies confirmed that miR-93 plays an oncogenic role in GC^78–80^. However, Stanitz et al. found no significant difference in the expression levels of miR-93 between GC tumor and normal tissues in the populations that they studied^81^.

The evaluation of correlation between different genes is important to explore the mechanisms leading to GC. We explored a strong positive correlation between β-catenin mRNA and miR-34a expression (Fig. 9). miRNAs regulate the expression of genes at the post-transcriptional and translational levels frequently by binding to complementary sequences in the 3′-UTRs of target mRNAs^79^. β-catenin, encoded by CTNNB1, is a predicted target for miR-34a in the databases such as miRTarbase (https://mirtarbase.mbc.nctu.edu.tw), mirDIP (https://ophid.utoronto.ca/mirDIP/), and TargetMinter (https://www.isical.ac.in/~bioinfo_miu/targetminer). The potential binding sites for miR-34a were located in the 3′-UTR of β-catenin mRNA (Supplementary Fig. 2) suggesting that β-catenin is a putative target of miRNA-34a. Furthermore, several studies experimentally demonstrated that β-catenin is a direct target of miR-34a which inversely affects the β-catenin mRNA and protein levels, as verified through luciferase reporter assay, immunoblot analysis and qRT-PCR^82–84^. Thus, our finding related to the positive correlation between miR-34a and β-catenin mRNA expression in GC tissues was in contrast to our primary assumption on the basis of previous studies. With further literature review, we found a study reporting that miR-34a expression was directly regulated by β-catenin and significantly induced by the overactivation of β-catenin signaling in mouse tumors and hepatocellular carcinoma patients^37^. However, a positive association between miR-34a and β-catenin, due to well-known oncogenic function of β-catenin as a co-transcriptional factor in Wnt/β-catenin signaling pathway, and common tumor suppressor function of miR-34a in majority of cancers, seems to be related to a little known mechanism, probably independent of p53 pathway. Nonetheless, we speculate that oncogenic function of miR-34a remains of matter of debate and needs to be clarified through further investigation.

Furthermore, we observed that the expression of β-catenin mRNA was positively correlated with the expression of miR-181a in GC tissues (Fig. 9). Consistent with this observation, we found a study by Ji et al. that reported a positive correlation between β-catenin expression and miR-181 family members in HCC (hepatocellular carcinoma) cell lines. In addition, they found that forced expression of β-catenin or Tcf4—a co-transcriptional activator of β-catenin—induced miR-181 expression^85^.

Moreover, we detected a concordant expression of miR-181a and miR-34a in GC tissues (Fig. 9), this may be due to the fact that both miR-34a and miR-181-a are transcriptional target of β-catenin. However, the confirmation of this finding needs to further research.

We detected a borderline significant positive correlation between the expression of β-catenin and GATA6 in GC tissues (*p* = 0.0603) (Fig. 9). GATA6 activates the expression of the wnt family members and wnt receptor Fzd2^86–89^. In addition, GATA6 negatively regulates the expression of Dickkopf-1 (DKK1), an inhibitor of Wnt signaling pathway^22^. These findings may illustrate a positive effect of GATA6 on β-catenin expression.

In previous reports, Notch1 was confirmed as direct target of miR-34a^90,91^. In this research, the inverse correlation between the RNA expression of miR-34a and Notch1 was not considerable (r = −0.1666; *p* = 0.4365). Furthermore, it was reported that miR-181a directly targets GATA6 and CDX2^92^. In our study, the expression of miR-181a was not noticeably correlated with that of GATA6 (r = 0.0591; *p* = 0.7836) and CDX2 (r = −0.2638; *p* = 0.2238). To elucidate the aforementioned findings, we note two important points in the following. Firstly, miRNAs as well as their targets could be cancer-specific. In support of this notion, Shi et al.^36^ suggested that the targets of the same miRNA in several cell lines of glioma may be different because each type of cell is likely to have a specific miRNA milieu for regulation of gene expression. Secondly, microRNAs directly suppress their target gene expression via mRNA degradation or translational repression^93^. Thus, to understand the precise roles of microRNAs in different malignancies, the evaluation of alterations at target protein level is beneficial in addition to that at mRNA expression level.

In the present study, there were no age-dependent differences in the expression patterns of seven candidate genes (data not shown). The majority of patients (22 of 24) included in the current study, were at TNM stage of III and IV. This prevented us from further investigation of possible differences in gene expression levels between early- and advanced stages. Moreover, no significant association was identified between the gene expression and clinical features of GC, including distant metastasis or between two intestinal- and diffuse-GC types (Figs. 8, 10). One explanation would be due to the extensive variations in expression levels between each individual patient (see Figs. 1, 2, 3, 4, 5, 6, 7 left).

## Conclusions

Despite the abundant research conducted to identify the molecular pathways implicated in GC pathogenesis, it remains largely unknown. The majority of GC patients are diagnosed in advanced stages due to poor prognosis of the disease. Thus, limited treatment options can be helpful for cure or improving survival of them. Therefore, there is an urgent need for robust treatment method. In this regard, the conduction of studies at molecular level would be more beneficial. However, it seems that complex nature of cancer gene-expression as well as the heterogeneity existing among the cancer subtypes or even between each individual patient are major obstacles affecting to achieve a better health outcome from the commonly used treatment procedures or methods that target a specific gene transcript/protein. On the other hand, the development of personalized medicine or targeting of the genes with prevalent dysregulation may lead to improve the health outcomes for individual patients after treatment. Nonetheless, further studies are required to identify the most prevalent molecular targets in GC patients.

Our study revealed that GATA6 expression was frequently decreased in GC patients. In addition, miR-93 expression was frequently increased in GC patients. We did not detect any correlation between the expression level of GATA6 and miR-93 (data not shown) implying these genes may affect on GC pathogenesis through two distinct signaling pathways. Notably, we determined a considerable positive correlation between β-catenin mRNA and miR-34a expression in GC tissues. Despite β-catenin was proved as a direct target of miR-34a, this finding may be attributed to the fact that miR-34a involves in a little known pathway that its induction occurs by β-catenin activity.

Overall, although the data presented in our study still needs to be proved by further studies, it may provide potential targets for the exploration of novel therapeutic strategies for GC treatment.

## Methods

### Collection of tissue samples

Twenty-four pairs of GC tissues and corresponding adjacent noncancerous gastric tissues (48 samples) were obtained from untreated patients who underwent routine endoscopy for diagnostic purposes at the following institutions placed in Sari, Iran: Tuba Clinic, Maziar Clinic, Imam Hospital, and Shafa Hospital. Biopsies were immediately placed in Fix RNA reagent (EURx, E0280, Gdańsk, Poland) and stored at 4 °C until RNA extraction that it was performed in less than one week. The clinical and histopathological parameters, such as gender, age, histological type, grade, and pathological stage were determined according to the medical reports of the patients and are summarized in Table 1. The study was approved by the Ethics Committee of the Mazandaran University of Medical Sciences and performed in accordance with the relevant guidelines. Informed consent was also obtained from patients prior to the study.

### RNA extraction and reverse transcription (RT)

Total RNA was extracted from tissue samples using Accuzol reagent (Bioneer, K-3090, Republic of Korea) according to the protocol. The concentration of total RNA was measured by UV spectrophotometry using a PicoDrop instrument. The extracted RNA was treated with RNase-free DNase I (Thermo Scientific, #EN0521) as described in the product manual to remove the possible contamination with genomic DNA.

Complementary DNA (cDNA) was synthesized from ~ 1 µg of DNase I-treated total RNA for each RT reaction using RevertAid First Strand cDNA synthesis kit (Thermo Scientific, #K1622) following the manufacturer’s instructions. Stem-loop RT primers were designed on the basis of Chen et al. study^94^ and used for the specific cDNA synthesis of miRNAs. Reverse transcription of U6 small non coding RNA—as internal control for the normalization of miRNA expression—was also performed using a specific primer taken from a published study^95^ (Supplementary Table 1). Equal volume (1:1) of oligo (dT)_18_ and random hexamer primers, provided by the kit, was mixed and used for cDNA synthesis from total RNA to quantify the expression of the other genes of interest in this study.

### Quantitative real-time PCR analysis

The expression level of β-catenin, Notch1, CDX2, GATA6, mature forms of miR-34a, miR-181a, and miR-93 was detected using quantitative RT-PCR. The qRT-PCR analysis was performed with SYBR Premix Ex Taq II (Takara, Japan) in an Exicycler 96 system (Bioneer, Republic of Korea). After an initial denaturation for 2 min at 95 °C, qRT-PCR was followed by 40–45 cycles at 95 °C for 15 s, and at 58 °C for 50 s. Relative quantification was based on the cycle threshold (Ct) values generated by the Exicycler3 Analysis software (Bioneer, Republic of Korea). U6 small nuclear RNA (snRNA) and Glyceraldehyde-3-phosphate-dehydrogenase (GAPDH) were used as internal control for the normalization of miRNA and mRNA expressions, respectively. The specificity of the amplified products was determined by electrophoresis of the qRT-PCR product on 2% agarose gel. All qRT-PCR reactions were run at least in duplicate and average Ct was calculated. The relative expression levels of the genes of interest were calculated by the 2^−∆∆Ct^ method, in which ΔΔCt = (Ct_miRNA/mRNA_ − Ct_U6/GAPDH_)_Tumor_ − median of (Ct_miRNA/mRNA_ − Ct_U6/GAPDH_)_Non Tumor_ and our results were represented as fold changes (FC) in expression of the genes in GC tissues relative to the non-cancerous tissues.

Primer sequences were either taken from published studies or designed (for miRs 34a, 181a, 93) (Supplementary Table 1). All the primers were synthesized by Bioneer company (Republic of Korea).

### Statistical analysis

To identify genes that were differentially expressed between the two groups, unpaired Mann–Whitney test was used. The statistical significance of correlation between the expression levels of the genes was evaluated by Spearman’s correlation test. The GraphPad Prism software version 6 (GraphPad Software Inc., CA, USA) was used for the statistical analysis and to generate graphs. Two‑tailed *p* values less than 0.05 were considered statistically significant differences (*p < 0.05; **p < 0.01; ***p < 0.001).

## Supplementary information

Supplementary Information.
