# Supplementary material for: Expression patterns of seven key genes, including β-catenin, Notch1, GATA6, CDX2, miR-34a, miR-181a and miR-93 in gastric cancer
Source: Sci Rep. 2020 Jul 23;10:12342. doi: 10.1038/s41598-020-69308-0 (PMC7378835; doi:10.1038/s41598-020-69308-0)
Supplement: Supplementary file 1 — Supplementary Information. [file 41598_2020_69308_MOESM1_ESM.docx]

**Expression patterns of seven key genes, including β-catenin, Notch1, GATA6, CDX2, miR-34a, miR-181a and miR-93 in gastric cancer**

**Narjes Jafari^1^, Saeid Abediankenari^1,*^, Zahra Hosseini-Khah^2^, Seyed Mohammad Valizadeh^3^, Zhila Torabizadeh^4^, Ehsan Zaboli^5^, Maryam Ghasemi^6^, Hafez Fakheri^3^, Vahid Hosseini^3^, Ramin Shekarriz^5^, Alireza Rafiei^7^, Hossein Asgarian-Omran^1^, and Fatemeh Abedian^8^**

^1^ Immunogenetics Research Center, Department of Immunology, Faculty of Medicine, Mazandaran University of Medical Sciences, Sari, Iran

^2^ Diabetes Research Center, Faculty of Medicine, Mazandaran University of Medical Sciences, Sari, Iran

^3^ Gut and Liver Research Center, Department of Internal Medicine, Faculty of Medicine, Mazandaran University of Medical Sciences, Sari, Iran

^4^ Department of Pathology, Faculty of Medicine, Mazandaran University of Medical Science, Sari, Iran

^5^ Gastrointestinal Cancer Research Center, Department of Internal Medicine, Faculty of Medicine, Mazandaran University of Medical Sciences, Sari

^6^ Immunogenetics Research Center, Department of Pathology, Faculty of Medicine, Mazandaran University of Medical Science, Sari, Iran

^7^ Molecular and Cell Biology Research Center, Department of Immunology, Faculty of Medicine, Mazandaran University of Medical Sciences, Sari, Iran

^8^ Student Research Committee, Mazandaran University of Medical Sciences, Sari, Iran

* Corresponding author: abedianlab@yahoo.co.uk

Supplementary data:

Table 1. Specific reverse transcription and quantitative real time PCR primers

| Product size (bp) in RT-PCR | Sequence (5ʹ-3ʹ) | Primer |
| --- | --- | --- |
| - | GTCGTATCCAGTGCAGGGTCCGAGGTATTCGCACTGGATACGACACTCAC | miR-181a-5p stem-loop (RT) |
| - | GTCGTATCCAGTGCAGGGTCCGAGGTATTCGCACTGGATACGACACAACC | miR-34a-5p stem-loop (RT) |
| - | GTC GTA TCC AGT GCA GGG TCC GAG GTA TTC GCA CTG GAT ACG ACCTACCT | miR-93-5p stem-loop (RT) |
| - | GAATTTGCGTGTCATCCTTG | U6 specific (RT) |
| ⁓70 | F: GGAAACATTCAACGCTGTCG  Universal R: GTGCAGGGTCCGAGGT | miR-181a-5p |
| ⁓70 | F: GGGATGGCAGTGTCTTAGC  Universal R: GTGCAGGGTCCGAGGT | miR-34a-5p |
| ⁓70 | F: GGACAAAGTGCTGTTCGTGC  Universal R: GTGCAGGGTCCGAGGT | miR-93-5p |
| 89 | F: GCTTCGGCAGCACATATACTAAAAT  R: CGCTTCACGAATTTGCGTGTCAT | U6 |
| 124 | F: CAATGTGGATGCCGCAGTTGTG  R: CAGCACCTTGGCGGTCTCGTA | Notch1 |
| 176 | F: GATACCTCCCAAGTCCTGTATGAG  R: GCATCAAACTGTGTAGATGGGATC | β- catenin |
| 125 | F: TCTACAGCAAGATGAACGGCCTCA  R: TCTGCGCCATAAGGTGGTAGTTGT | GATA6 |
| 179 | F: AGACCAACAACCCAAACAGC  R:CCCGAACAGGGACTTGTTTA | CDX2 |
| 113 | F: CATGAGAAGTATGACAACAGCCT  R: AGTCCTTCCACGATACCAAAGT | GAPDH |

F, forward; R, reverse; RT, reverse transcription

**Fig. 1.** Correlation patterns of the genes in gastric cancer tissues examined using quantitative RT- PCR. Curves show the correlations between the expression of the genes in 15 intestinal- and 9 diffuse-type gastric cancer tissues. Expression levels are given as Log_2_^Fold Change^ (or -∆∆ ct). R and P-values were calculated by Spearman ʼs correlation method. *, and ** indicate P < 0.05, and P < 0.01, respectively.


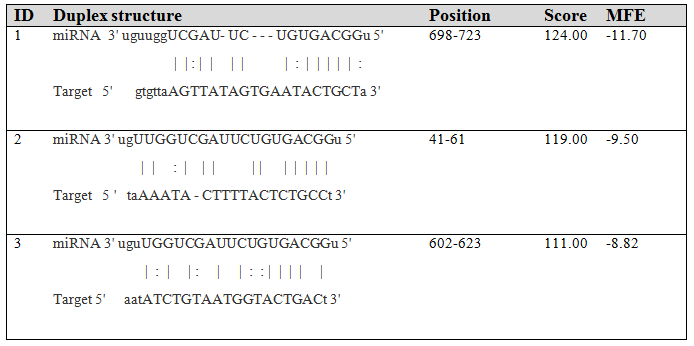


**Fig 2**. Complementary sequence interaction between miR-34a-5p and CTNNB1. Potential binding sites of miR-34a-5p in β-catenin mRNA (NM_001904) 3ʹ UTR and miRNA-target interactions are shown, as predicted by miRanda and presented by miRtarBase (http://mirtarbase.mbc.nctu.edu.tw). MFE; Minimum free energy**.**
